# Overexpression of LASS2 inhibits proliferation and causes G0/G1 cell cycle arrest in papillary thyroid cancer

**DOI:** 10.1186/s12935-018-0649-1

**Published:** 2018-10-01

**Authors:** Feng Zeng, Liangliang Huang, Xiaoming Cheng, Xiaoli Yang, Taolang Li, Guoli Feng, Yingqi Tang, Yan Yang

**Affiliations:** 1 Medical Center of Breast and Thyroid Disease, Affiliated Hospital of ZunYi Medical College, Zunyi, 563003 Guizhou People’s Republic of China; 2 College of Laboratory Medicine, Affiliated Hospital of ZunYi Medical College, Zunyi, 563003 Guizhou People’s Republic of China; 3 Department of Clinical Laboratory, Affiliated Hospital of ZunYi Medical College, 149 Dalian Road, Zunyi, 563003 Guizhou People’s Republic of China

**Keywords:** LAG1 longevity-assurance homologue 2, Papillary thyroid cancer, Proliferation, Cell cycle

## Abstract

**Background:**

The aim of this study was to investigate the role of LAG1 longevity-assurance homologue 2 (LASS2) in papillary thyroid cancer (PTC).

**Methods:**

Immunohistochemistry staining was conducted to explore the expression levels of LASS2 in PTC tissues and adjacent normal thyroid tissues and nodular goiter tissues. Western blotting and RT-qPCR were performed to explore the expression levels of LASS2 in three PTC cell lines (TPC-1, K1, BCPAP). An Adv-LASS2-GFP recombinant adenovirus vector was constructed and transduced into BCPAP cells. Then CCK-8 assay, colony formation assay, cell cycle distribution, and apoptosis were performed. Western blotting was used to examine the expression of p21, cyclin D1, cyclin-dependent kinase 4, p53 and p-p53.

**Results:**

LASS2 was downregulated in PTC tissues compared with adjacent thyroid tissues or nodular goiter tissues. In addition, the expression of LASS2 was found to be associated with TNM stage and lymph node metastasis. BCPAP cells expressed the lowest LASS2 compared to TPC-1 cells or K1 cells. Overexpression of LASS2 significantly inhibited proliferation, promoted apoptosis and caused G0/G1 cell cycle arrest in BCPAP cells. Furthermore, overexpression of LASS2 significantly increased the expression of p21, inhibited the expression of cyclin D1 and cyclin-dependent kinase 4, and increased the expression of p-p53, but did not effect the expression of p53 in BCPAP cells.

**Conclusion:**

Our findings indicate that overexpression of LASS2 inhibits PTC cell proliferation, promotes apoptosis and causes G0/G1 cell cycle arrest via a p53-dependent pathway. Thus, LASS2 may serve as a novel biomarker in PTC.

## Background

Thyroid cancer is the most common endocrine tumor, accounting for 96% of all endocrine neoplasms and 66.8% of all cases of endocrine tumor-related deaths [1]. In the United States, the incidence of thyroid cancer is increasing, while that of several head and neck tumors is decreasing [2]. Interestingly, the increase in the incidence of thyroid cancer is mainly due to increased cases of papillary thyroid cancer (PTC), while the increase in follicular or medullary subtypes is significantly less prominent [3]. It was previously demonstrated that the molecular mechanisms of thyroid cancer mainly involve genetic and epigenetic changes, such as gene mutations, leading to the activation of oncogenes and the inactivation of tumor suppressor genes [4]. With the development of molecular biology technology, researchers have identified a variety of oncogenes and tumor suppressor genes that may serve as molecular markers in thyroid tumors, such as BRAF, PTEN, CRABP1, C1QL1 and LCN2 [5–7]. In recent years, there has been a general consensus regarding molecular markers as potential diagnostic and prognostic tools for thyroid cancer. Krishnamurthy et al. [8] reported that the incorporation of novel molecular markers in patients with conventional PTC is of great significance in diagnosis and treatment individualization. However, the molecular mechanisms that have already been identified are unable to explain 100% of the cases.

LAG1 longevity assurance homolog 2 (LASS2), also referred to as ceramide synthase 2 (CerS2), was designated as LASS2 by researchers at Shanghai Fudan University in 2001 [9]. LASS2 belongs to the family of mammalian CerS genes involved in sphingolipid metabolism, and is known as a tumor metastasis suppressor [10, 11]. Previous studies have demonstrated that the LASS2 gene is associated with the proliferation, invasion, metastasis and apoptosis of multiple cancer cells, such as bladder, prostate, breast and liver cancer cells [12–15], and is associated with worse prognosis of meningiomas and bladder cancer [16, 17]. Therefore, it may represent a potential biomarker for several cancers. Jin et al. [18] reported decreased formation of the cyclin-dependent kinase (CDK)4/cyclin D1 complex following partial hepatectomy in liver-specific LASS2 knockout mice, indicating that it may play a role in cell cycle regulation. In addition, Su et al. [19] reported that overexpression of LASS2 inhibited cell proliferation by mediating the induction of G0/G1 cell cycle arrest in 293 and 293T cells, while Fan et al. [20] revealed that the overexpression of LASS2 had no effect on cell cycle progression in the breast cancer cell line MCF-7/ADR. However, the potential molecular mechanism underlying LASS2-mediated biological behavior in various types of cancer remains elusive. Previous studies have focused on the regulation of LASS2 in the tumor microenvironment, and revealed that it may inhibit cancer invasion and metastasis by regulating V-ATPase activity and extracellular H^+^ concentration [12]. However, to the best of our knowledge, there has been no report investigating the role of LASS2 in thyroid cancer to date.

The aim of the present study was to analyze the role of LASS2 in thyroid cancer using patient samples and the PTC cell line BCPAP, in order to determine whether overexpression of LASS2 inhibits PTC cell proliferation and elucidate the underlying mechanism, hoping to identify a new potential molecular marker in this type of cancer.

## Methods

### Patients and tissue samples

Thyroid tissues were obtained from 97 patients at the time of initial surgery at the Medical Center of Breast and Thyroid Disease of the Affiliated Hospital of Zunyi Medical College (Guizhou, China) and stored at − 80 °C. The study protocol was approved by the Ethics Committee of the Affiliated Hospital of Zunyi Medical College, and human thyroid tissues were obtained following provision of informed consent by the patients. All tissue samples were examined and the diagnosis was confirmed by two pathologists.

### Cell lines and cell culture

The BCPAP, TPC-1 and K1 cell lines were provided by Stem Cell Bank, Chinese Academy of Sciences (Shanghai, China) and cultured in RPMI-1640 supplemented with 10% FBS and incubated at 37 °C in a 5% CO_2_ atmosphere.

### Reverse transcription-quantitative polymerase chain reaction (RT-qPCR) analysis

Total RNA was extracted from BCPAP cells using TRIzol reagent (Takara Bio Inc., Otsu, Japan), according to the RNAiso Plus kit instructions. Total RNA was reverse-transcribed to cDNA using Prime ScriptTMRT reagent kit (Takara Bio Inc.). RT-qPCR was performed using SYBR^®^Premix Ex TaqTM II (Takara Bio Inc.). GAPDH was used as endogenous control. The primers for RT-qPCR were as follows: LASS2: forward, 5′-ATCGTCTTCGCCATTGTT-3′ and reverse, 5′-CGGTCACTGCGTTCATCT-3′; GAPDH: forward, 5′-GGAGCGAGATCCCTCCAAAAT-3′ and reverse, 5′-GGCTGTTGTCATACTTCTCATGG-3′ (197 bp). The levels of LASS2 were analyzed by the 2^−∆∆Ct^ method.

### Immunohistochemistry

Paraffin-embedded specimens were cut into 3-μm sections for hematoxylin and eosin (H&E) staining and immunohistochemistry (IHC). The assays were performed as previously described [16], and the primary antibody used was anti-LASS2 at a dilution of 1:200 (Abcam, Cambridge, UK).

The scoring of LASS2 expression was also performed as previously described [16]. The intensity distribution (ID) score for LASS2 was evaluated by the sum of the percentage of positive cells (0: < 5%; 1: 5–25%; 2: 26–50%; 3: 51–75%; and 4: 76–100%) and the staining intensity was graded from 0 to 3 (0, negative; 1, weak; 2, moderate; and 3, strong). ID scores ≥ 6 were considered to reflect high expression, and those ≤ 4 low expression.

All histological stainings were evaluated by two pathologists, and the scores were calculated by two observers.

### Construction of Adv-LASS2-GFP recombinant adenovirus vector

The LASS2 recombinant eukaryotic expression of LASS2-GFP (as insert segment) and pShuttle-CMV recombinant shuttle vector, were constructed and digested with *Hin*dIII/*Not*I, respectively, and then ligated with T4 DNA ligase. Subsequently, transformed plasmids were extracted and sequencing was performed to obtain pShuttle-LASS2-GFP recombinant shuttle plasmid. Next, the pAdxsi vector and pShuttle-LASS2-GFP were separately digested with I-Ceul and I-Scel, then ligated and transformed. To obtain pAdxsi-LASS2-GFP viral plasmid, extensive extraction of the viral plasmids was performed, followed by packaging, collection and amplification. Finally, a recombinant Adv-LASS2-GFP was obtained, with a titer of 1.2 × 10^10^ PFU/ml.

### Western blotting

The BCPAP cells were lysed with RIPA lysis buffer (1% NP-40, 0.1% SDS, 50 mM DTT) containing protease inhibitor cocktail on ice. After centrifugation, the supernatant was collected in 1.5-ml centrifuge tubes. The cell lysate was loaded on 10% sodium dodecyl sulfate polyacrylamide (SDS-PAGE) gels after being heated at 100 °C for 3 min for denaturation and then transferred onto PVDF membranes. The membranes were blocked with 5% skimmed milk at 37 °C for 2 h. The blocked membranes were washed with PBST buffer 2–3 times and incubated with the primary antibodies for 2 h at room temperature. After the membranes were washed four times with PBST buffer, they were incubated with a corresponding secondary antibody in PBST buffer at 4 °C overnight, followed by washing four times with PBST. The blots were detected using Enhanced Chemiluminescence Detection kit (KGP116, KeyGen BioTECH, Jiangsu, China). The primary antibodies used in the experiment were anti-p53 (ab31333, 1:1000), anti-CDK4 (ab137675, 1:2000), anti-cyclin D1 (ab137875, 1:5000) and p21 (ab109520, 1:1000), all purchased from Abcam; Anti-p-p53 (#9284, 1:1000) was purchased from CST; Anti- GAPDH (SC-365062, 1:800) was purchased from Santa Cruz Biotechnology, Inc. (Dallas, TX, USA). The secondary antibody used in the experiment were: goat anti-rabbit, goat anti-mouse IgG (1:4000 for both).

### Subcellular localization of LASS2 by confocal laser scanning microscopy in BCPAP cells

BCPAP cells were transfected with Adv-LASS2-GFP for 48 h. After washing three times with PBS, the cells were fixed with 4% paraformaldehyde for 30 min. Autofluorescence from the LASS2 protein was observed under a confocal microscope (LSM510; Carl Zeiss AG, Oberkochen, Germany) with excitation at 488 nm and a 525-nm GFP filter. Images were acquired at a ×200 magnification.

### Cell proliferation assay

For the proliferation assay, BCPAP cells were treated with Cell Counting Kit-8 reagent (Beyotime Institute of Biotechnology, Shanghai, China), according to the manufacturer’s instructions. Absorbance at 450 nm was measured on a microplate reader at the designated time points after treatment. The assay was performed in triplicate.

### Colony formation assay

For the colony formation assay, the transfected cells were plated at 1000 cells/well in a 6-well plate and incubated for 2 weeks. The colonies were fixed and then stained with 0.5% (w/v) crystal violet solution for 30 min at room temperature. The colonies were photographed and counted. The assay was performed in triplicate.

### Cell cycle analysis

BCPAP cells transfected with Adv-GFP or Adv-LASS2-GFP were harvested 48 h after culture, washed with PBS and fixed in 500 µl of 75% cold ethanol at 4 °C overnight, and then washed with PBS, stained by 100 µl propidium iodide (3.8 × 10^−2^ sodium citrate, pH 7.0) containing RNase A (10 mg/ml) for 30 min in the dark at 37 °C. The cell populations in the G0–G1, S and G2-M phases were measured by flow cytometry (Beckman Coulter, Inc., Brea, CA, USA), and analyzed using ModiFit software. All the samples were assayed in triplicate.

### Annexin-V APC/7-AAD double-staining to detect apoptosis

Following transfection with Adv-GFP or Adv-LASS2-GFP, BCPAP cells were harvested with 0.25% trypsin (without EDTA). Complete medium was added to the cells to inactive trypsin, and the cells were washed twice with PBS (centrifugation at 800*g*, 5 min). The cells were subsequently resuspended in 500 µl of binding buffer. After 5 µl of Annexin V-APC was added and mixed well, 5 µl of 7-AAD was added and mixed well. The cells were incubated at room temperature for 15 min in the dark, then immediately analyzed using a flow cytometer (FACSCalibur, BD Biosciences, San Diego, CA, USA) using CellQuest software.

### TUNEL assay

The amount of DNA fragmentation was determined using a commercial BIOTIN labeling dUTP TUNEL kit (KeyGen BioTECH, Jiangsu, China) according to the instructions of the manufacturer. Briefly, BCPAP cells on the slides were washed with PBS three times, and then fixed with 4% paraformaldehyde for 30 min, permeabilized with 1% Triton X-100 at room temperature for 15 min, and then treated with 3% H_2_O_2_ containing methanol for 15 min. After the enzymatic reaction, cells were washed with PBS, incubated with a mixture of TdT solution and fluorescein isothiocyanate dUTP solution at 37 °C for 60 min in a humidified chamber, then incubated with 100 µl streptavidin–horseradish peroxidase at 37 °C for 30 min in a humidified chamber. After washing with PBS, the cells were stained with DAB solution, followed by counterstaining with hematoxylin, and observed under a light microscope. Cells with brown granules in the nuclei were considered as TUNEL-positive. Cells were counted (high-power field, magnification ×200) under an optical microscope (Olympus, Tokyo, Japan) to determine the mean percentage of positive cells.

### Statistical analysis

All statistical analyses were performed using SPSS 21.0 software (SPSS version 21.0., IBM Corp., Armonk, NY, USA). GraphPad Prism 5 (GraphPad Software Inc., San Diego, CA, USA) was used for graphs. The Chi squared test or Fisher’s exact test was used to evaluate the association between clinicopathological characteristics and LASS2 expression. Student’s t-test was performed to analyze differences between groups. All values represent at least three independent experiments and are expressed as the mean ± standard deviation. *P *< 0.05 was considered to indicate statistically significant differences.

## Results

### Expression of LASS2 in thyroid tissues

**Fig. 1:**
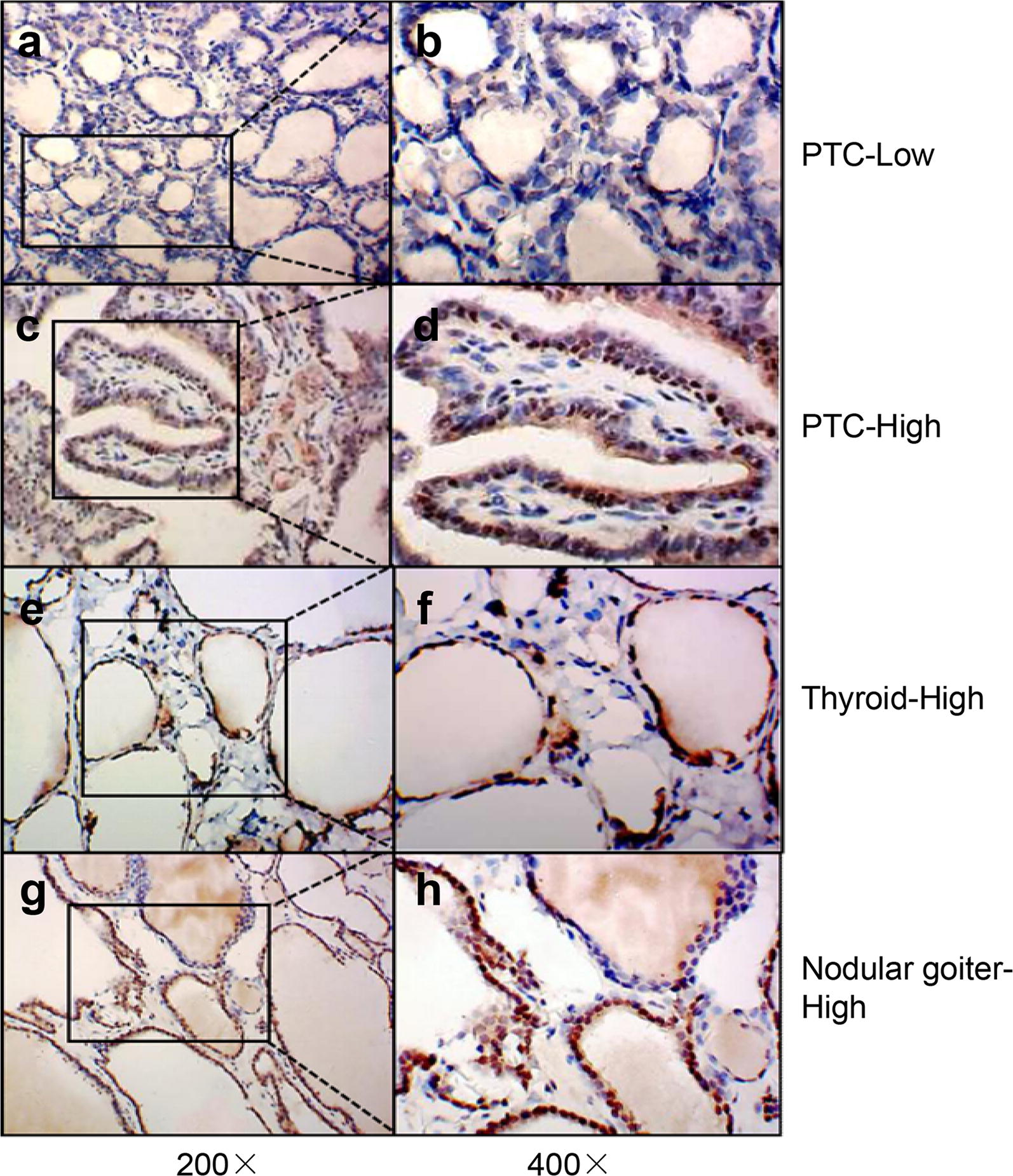
Immunohistochemical analysis of LASS2 expression in PTC, adjacent thyroid tissues and nodular goiter. **a**–**d** Representative immunohistochemical staining patterns for LASS2 in PTC. Its protein expression level was graded as low, high. **e** and **f** Representative immunohistochemical staining patterns for LASS2 in adjacent thyroid tissues. Its protein expression level was graded as high. **g** and **h** Representative immunohistochemical staining patterns for LASS2 in nodular goiter. Its protein expression level was graded as high. The LASS2 expression was located in cytoplasm and/or nucleus. Left and right panels were ×200 and ×400 amplification, respectively

To explore the LASS2 expression in thyroid tissues, IHC was performed in 117 paraffin-embedded specimens, including 60 PTC, 20 adjacent thyroid tissues and 37 nodular goiters. LASS2 immunoreactivity was mainly detected in the cytoplasm and nucleus (Fig. 1).

To validate the expression LASS2 in different thyroid tissues, immunostaining was performed by semi-quantitative analysis and represented by an ID score (Table 1). The results revealed that the number of cases scoring ≥ 6 was 14 (14/20, 70.00%) in adjacent thyroid tissues, 21 (21/37, 56.76%) in nodular goiter tissues, and 23 (23/60, 38.33%) in PTC tissues. These results indicate that LASS2 expression was decreased in PTC.

**Table 1:** Correlations of LASS2 expression with different thyroid tissues (n = 117)

Thyroid tissues	LASS2 ID score		*P*
	0–4	6–12	
PTC	37	23	0.028*
Adjacent thyroid tissues	6	14	
nodular goiter	16	21	

### Correlations between LASS2 expression and clinicopathological parameters

To determine whether LASS2 expression is associated with tumorigenesis and progression of PTC, we investigated the correlations between this gene and clinicopathological parameters (Table 2). It has been observed that the LASS2 expression level was positively correlated with TNM stage and lymph node metastasis (LNM) (*P *= 0.045 and 0.001, respectively). However, there were no observed correlations between LASS2 expression and patient sex, age, tumor size or location (all *P *> 0.05).

**Table 2:** Association between the LASS2 protein and high-risk characteristics in patients with papillary thyroid cancer (n = 60)

Characteristics	LASS2 ID score		*P*
	0–4	6–12	
Sex			0.142
Male	7	8	
Female	30	15	
Age (years)			0.354
< 45	21	15	
≥ 45	16	8	
Tumor size			0.067
≤ 1 cm	8	10	
> 1 cm	29	13	
TNM Stage			0.045*
I	23	21	
II	1	1	
III	4	1	
IV	9	0	
Location			0.128
Unilateral	28	21	
Bilateral	9	2	
LNM			0.001*
Negative	6	14	
Positive	31	9	

### The expression and subcellular localization of LASS2 in PTC cell lines

**Fig. 2:**
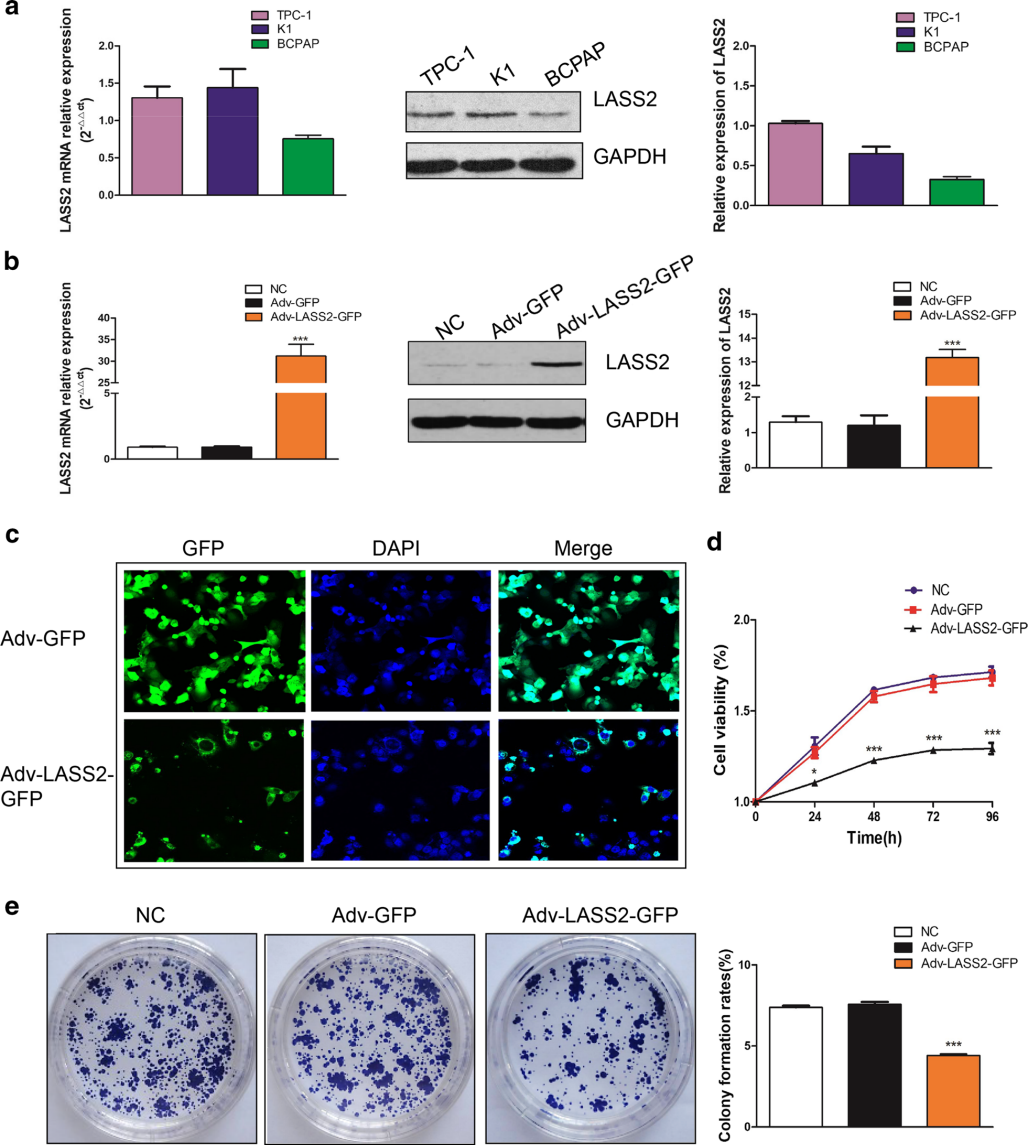
The expression of LASS2 in PTC cell lines and overexpression of LASS2 inhibits the proliferation of BCPAP cells. **a** The mRNA and protein levels of LASS2 in three PTC cell lines. × The mRNA and protein levels of LASS2 in Adv-GFP or Adv-LASS2-GFP transfected BCPAP cells. ***P < 0.001. **c** Subcellular localization of Adv-LASS2-GFP in BCPAP cells. **d** CCK-8 assays in up-regulation BCPAP cells and their corresponding control cells.*P < 0.05; ***P < 0.001. **e** Colony formation assays in up-regulation BCPAP cells and their corresponding control cells. ***P < 0.001 in comparison with the NC or vector group using Student’s t-test. All the experiments were performed at least three times

To investigate the biological function of LASS2, we detected its expression in three PTC cell lines (TPC-1, K1, BCPAP) (Fig. 2a). The results showed that BCPAP cells expressed the lowest LASS2 compared to TPC-1 cells or K1 cells. Then we successfully overexpressed LASS2 in BCPAP cells using adenovirus vectors (Fig. 2b). We further examined the subcellular distribution of LASS2 in BCPAP cells using a confocal laser scanning microscope. Consistent with the results of IHC, the results demonstrated that LASS2 mainly localized to the cytoplasm and nucleus, with a lower distribution to the cell membrane (Fig. 2c).

### Overexpression of LASS2 inhibits the proliferation of BCPAP cells

In order to determine the role of LASS2 in BCPAP cells, CCK-8 and colony formation assays were performed. It was clearly demonstrated that upregulated LASS2 expression effectively inhibited BCPAP cell proliferation and colony formation compared with the corresponding control cells (Fig. 2d, e, both *P *< 0.05).

### Overexpression of LASS2 causes cell cycle arrest at the G0/G1 phase in BCPAP cells

**Fig. 3:**
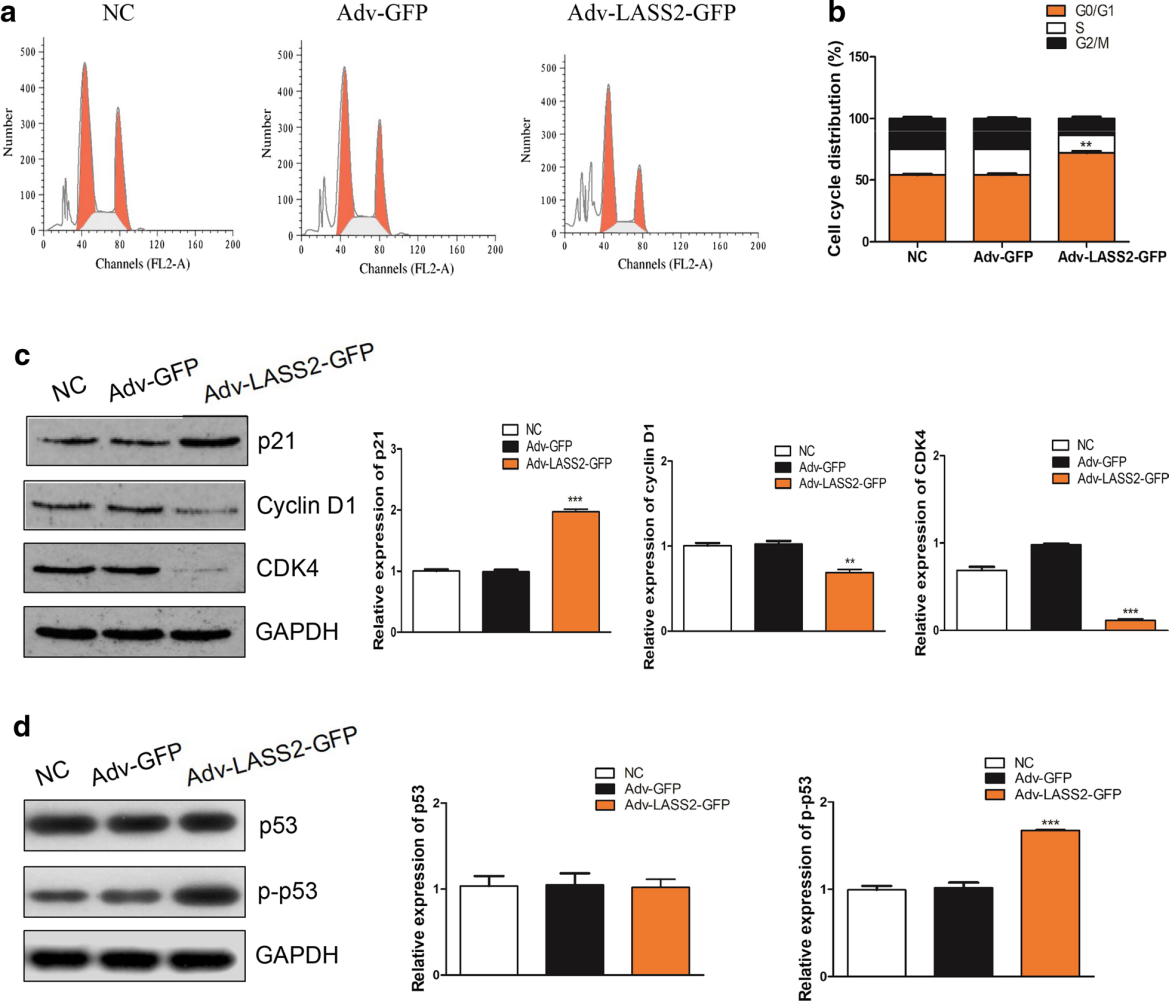
Overexpression of LASS2 inhibits cell cycle progression in BCPAP cells. **a** and **b** Flow-cytometry analysis showed that overexpression of LASS2 significantly causes G0/G1 phase cell cycle arrest in BCPAP cells. **P < 0.01. **c** Effect of LASS2 on cell cycle-related gene expression was determined by western blotting. **P < 0.01; ***P < 0.001. **d** Effect of LASS2 on p-p53 expression. ***P < 0.001. All the experiments were performed at least three times

We used cell-based cytometry and western blotting to evaluate whether LASS2 regulates the cell cycle progression in BCPAP cells treated with Adv-GFP or Adv-LASS2-GFP. LASS2 induced arrest at the G0/G1 phase in BCPAP cells after 48 h of treatment (Fig. 3a, b, *P *< 0.01); the percentage of cells in the G0/G1 phase in the NC, Adv-GFP and Adv-LASS2-GFP groups was 54.01, 54.13 and 71.94%, respectively.

Furthermore, it was demonstrated that LASS2 significantly upregulated p21, whereas it downregulated CDK4 and cyclin D1 (Fig. 3c, all *P *< 0.05). However, the p53 protein, which regulates the p21 gene [21], was not found to be significantly up- or downregulated (Fig. 3c, *P *> 0.05). Furthermore, in order to evaluate the role of p53 better, we detected the expression of p-p53 and found it was efficiently increased (Fig. 3d, *P *< 0.001), pointing to a p53-dependent pathway for p21 activation in cell cycle progression and G0/G1 phase arrest.

### Overexpression of LASS2 promotes apoptosis of BCPAP cells

**Fig. 4:**
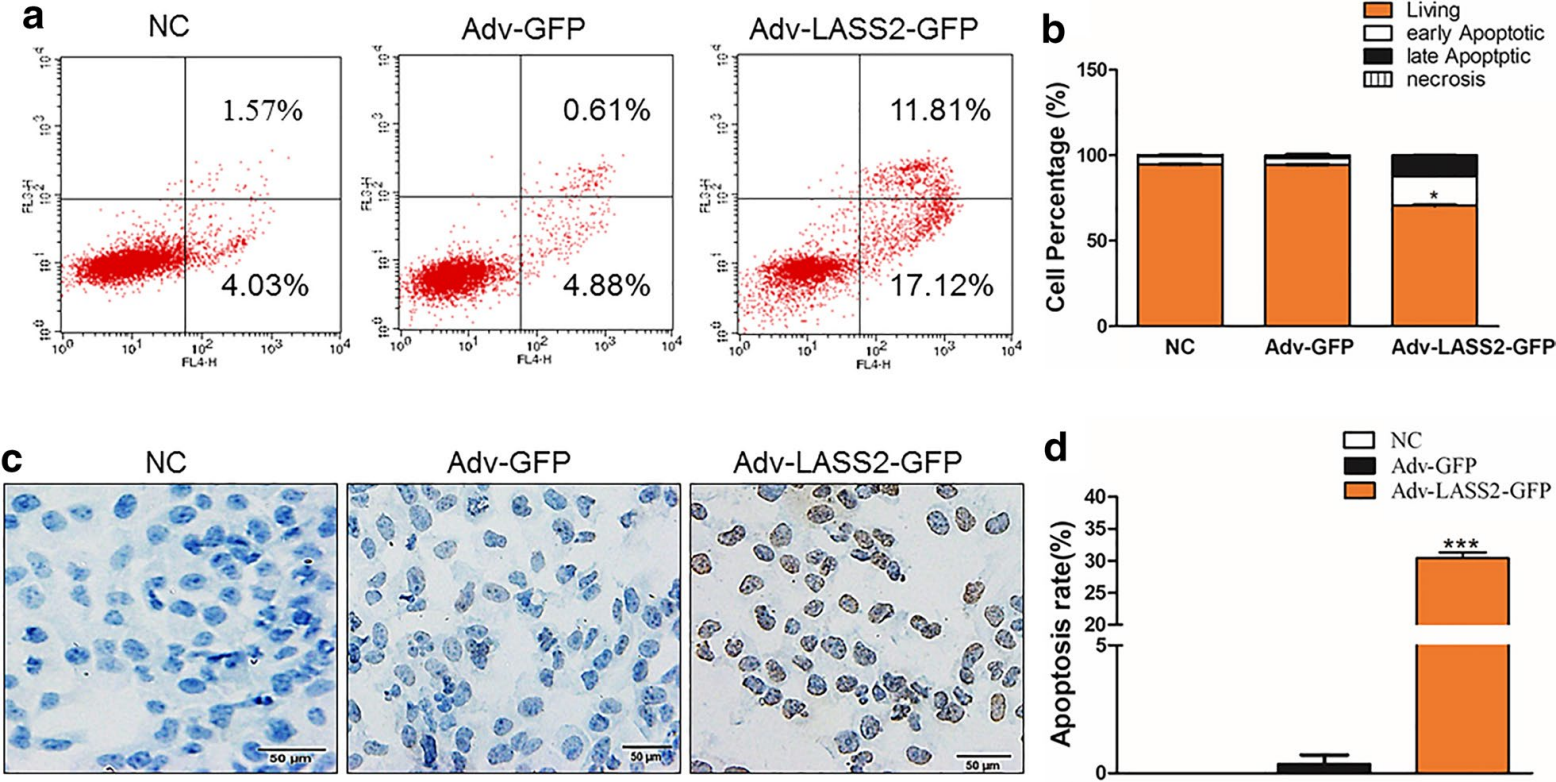
Overexpression of LASS2 affects cell apoptosis in BCPAP cells. **a** FACS sorting of BCPAP cells treated with either Adv-LASS2-GFP or Adv-GFP. **b** Quantification of living, early apoptotic, late apoptotic, or necrotic BCPAP cells. *P < 0.05. **c** Apoptosis was detected using TUNEL staining for the BCPAP cells treated with different treatments. **d** The apoptosis rate of BCPAP cells treated with different treatments, ***P < 0.001. All the experiments were performed at least three times

As shown in Fig. 4, overexpression of LASS2 significantly affected the apoptosis of BCPAP cells. An Annexin V-APC/7-AAD kit was used for these assays, and the results indicated that the apoptotic index of LASS2-infected cells (28.93%) was markedly higher compared with that of negative control cells (5.49%) (Fig. 4a, b).

To further determine whether apoptosis induction in BCPAP cells was attributable to LASS2, a TUNEL assay was performed. Consistent with the results of Annexin-V APC/7-AAD double-staining, the apoptosis rate of BCPAP cells treated with Adv-LASS2-GFP (30.41%) was markedly increased compared with the Adv-GFP treatment (0.36%) or no treatment groups (0.00%) (Fig. 4c, d).

## Discussion

Sphingolipids have been identified as bioeffector molecules, controlling various aspects of cell proliferation in cancer [22]. LASS2 is a known regulator of sphingosine levels [23], and generally mediates antiproliferative responses, such as cell cycle arrest and apoptosis induction.

Rong-Hu et al. [16] reported a high correlation between LASS2 expression and Ki-67 in meningiomas, indicating their potentially similar function in tumorigenesis as proliferation-related proteins. This is in agreement with our results in human thyroid tissues, demonstrating that LASS2 was downregulated in PTC compared with adjacent non-cancerous tissues or benign disease in 97 patients. To the best of our knowledge, our study is the first to demonstrate that LASS2 was downregulated in PTC. Clinicopathological characteristics analysis revealed that the LASS2 expression level was positively correlated with TNM stage and LNM (both *P *< 0.05). However, there were no observed correlations between LASS2 expression and patient sex, age, tumor size or location (all *P *> 0.05).

In order to better evaluate the role of LASS2 in thyroid cancer, we detected its expression in three PTC cell lines and found that BCPAP cells expressed the lowest LASS2 compared to TPC-1 cells or K1 cells. Then we successfully overexpressed LASS2 in BCPAP cells using adenovirus vectors. Consistent with the results of IHC, our results demonstrated that LASS2 mainly localizes to the cytoplasm and nucleus, and less prominently to the cell membrane. Furthermore, overexpression of LASS2 significantly inhibited PTC cell proliferation.

Resisting cell death and sustained proliferation are considered to be the fundamental hallmarks of cancer [24]. Dysregulation of cell cycle progression subverts the dynamic balance of cell proliferation and cell death, thereby leading to cancer development [25, 26]. Previous studies have demonstrated that the use of ceramide analogues or mimics may promote apoptosis and cause cell cycle arrest at the G0/G1 phase in cancer cells [27, 28]. Su et al. [19] reported TMSG1/LASS2 as a potential metastasis suppressor gene, involved in the induction of apoptosis and G0/G1 cell cycle arrest in 293 and 293T cells. This is consistent with our in vitro data. In the present study, we confirmed that overexpression of LASS2 promoted cell apoptosis and caused G0/G1 arrest in BCPAP cells. Furthermore, we investigated the potential mechanisms involved in cell cycle arrest. The cell cycle is controlled by protein kinase complexes consisting of cyclins and cyclin-dependent kinases (CDKs) [29]. In G1 phase, D-type cyclins (D1, D2 or D3) bind to CDK4 and CDK6 and control the transition through key checkpoints, after which the cell cycle can proceed autonomously [30]. Previous studies have uncovered that the transcription factor cellular tumor antigen p53 regulates the expression of numerous genes involved in the cell cycle and induces cell cycle arrest [31–33]. P21, a general G1 phase cell cycle inhibitor, was the first p53-effector gene [34]. In the present study, we demonstrated that the overexpression of LASS2 resulted in the downregulation of cyclin D1 and CDK4, induced the expression of p21, and increased the expression of p-p53, but did not significantly affect the expression of p53. Taken together, these results suggested that LASS2 overexpression induced G0/G1 cell cycle arrest may via a p53-dependent pathway. However, p21 was also known to be regulated by p53-independent signaling pathway [35, 36]. During the synthesis of phospho-sphingolipids, the generation of diacylglycerol and sphingosine-1-phosphate is necessary for the G1-S transition of cell cycle [37]. Sphingolipid pathway-mediated the activation of the NF-κB [38]. Nicolae et al. [39] have identified a novel NFκB-mediated mechanism of p53-independent activation of p21. Previous studies has showed that NF-κB regulated cell cycle target gene Cyclin D1 [40, 41]. Furthermore, Schumm et al. [42] found that NF-κB stimulated the expression of Cyclin D1 and repressed the expression of p21, revealed a role for NF-κB as regulator of cell cycle. Therefore, it may be not only p53, but also NF-κB, that mediates these regulatory effects. The mechanisms underlying the role of LASS2 in BCPAP cell cycle regulation require further investigation.

## Conclusion

Our experimental data revealed that LASS2 is downregulated in PTC tissues compared with adjacent non-cancerous tissues or benign diseases. To the best of our knowledge, this is the first study to demonstrate that LASS2 overexpression inhibited the proliferation ability, facilitated apoptosis and caused G0/G1 cell cycle arrest via a p53-dependent pathway in BCPAP cells. Thus, LASS2 may represent a new potential marker for the diagnosis and treatment of PTC.

## Abbreviations

LASS2: LAG1 longevity-assurance homologue 2
PTC: papillary thyroid cancer
CerS2: ceramide synthase 2
CDK4: cyclin-dependent kinase 4
H&E: hematoxylin and eosin
IHC: immunohistochemistry
ID: intensity distribution
SDS-PAGE: sodium dodecyl sulfate polyacrylamide
